# RHO Family GTPases in the Biology of Lymphoma

**DOI:** 10.3390/cells8070646

**Published:** 2019-06-26

**Authors:** Claudia Voena, Roberto Chiarle

**Affiliations:** 1 Department of Molecular Biotechnology and Health Sciences, University of Torino, 10126 Torino, Italy; 2 Department of Pathology, Boston Children’s Hospital and Harvard Medical School, Boston, MA 02115, USA

**Keywords:** RHO family GTPases, RHOA, RHOH, VAV, mutations, chromosomal translocations, lymphoma

## Abstract

RHO GTPases are a class of small molecules involved in the regulation of several cellular processes that belong to the RAS GTPase superfamily. The RHO family of GTPases includes several members that are further divided into two different groups: typical and atypical. Both typical and atypical RHO GTPases are critical transducers of intracellular signaling and have been linked to human cancer. Significantly, both gain-of-function and loss-of-function mutations have been described in human tumors with contradicting roles depending on the cell context. The RAS family of GTPases that also belong to the RAS GTPase superfamily like the RHO GTPases, includes arguably the most frequently mutated genes in human cancers (*K-RAS*, *N-RAS*, and *H-RAS*) but has been extensively described elsewhere. This review focuses on the role of RHO family GTPases in human lymphoma initiation and progression.

## 1. Introduction

RHO GTPases are highly conserved proteins in eukaryotes, and the RHO family GTPase consists of 18–22 members [1,2,3]. They can be classified according to their homology and structure into the following subfamilies: CDC42, RAC, RHO, RHOD, RHOU, RHOH, and RND [1,2,4,5]. RHOBTB subfamily is included in some studies, but they are quite divergent from the other subfamilies. Based on their structure, regulation, and function they are further classified as “typical” or “atypical”, where CDC42, RAC1, and RHOA are considered the prototype GTPases. Atypical GTPases diverge from the main family for amino acid substitutions in the RHO domain that alter their function. Most Rho GTPases are expressed ubiquitously, while others like RAC2 and RHOH have tissue-specific expression in the hematopoietic cells. Both typical and atypical RHO GTPases have been implicated in human cancer [1,2].

Rho family GTPases have a central role in a wide range of cellular processes [6,7]. Initially they were known as regulators of cytoskeleton remodeling, and thus linked to cell shape, cell polarity, cell adhesion and motility, and vesicle trafficking [8]. RHOA, RAC1, and CDC42, the best studied members of the family, were first described for their specific functions in promoting the formation of stress fibers, lamellipodia and filopodia in fibroblasts during actin cytoskeleton reorganization [4,9]. Later on, they were associated with other fundamental cellular processes, such as cell cycle progression and cell survival [10]. In addition, they were described to be involved in cell-specific processes, such as immune response [11], angiogenesis [12], and neurogenesis [13]. Alterations of RHO family GTPases and their related pathways contribute to a large variety of diseases, including malignant transformation and cancer progression [2,14,15,16,17]. Their role in cancer has been debated for a long time, since most of the information came from in vitro studies. Constitutive active RHOA (G14V) and RAC1 (G12V) mutants were described to have transforming properties in fibroblasts, although weakly than RAS oncogenes and for long time they were related to cancer only through their cooperative role in RAS or other oncogene-mediated transformation [2,18,19,20,21,22]. The recent identification of point mutations in the RHO GTPases, RAC1, RHOA, and CDC42 [23], or in their regulators, for example the RHO GEF VAV1 [24,25], or the demonstration of different expression levels in human tumors using high-throughput genomic analysis, have finally shown that RHO GTPases have a direct role in tumorigenesis.

Both gain- and loss-of-function mutations in *RHO* genes have been reported in human tumors as well as the overexpression and suppression of some members of the RHO family or of their regulators, as reviewed in [1]. To date the most frequent mutations in human cancer have been found in *RAC1* and *RHOA* genes, whereas very few mutations have been found in *CDC42* gene [1,16]. A recurrent gain-of-function mutation in *RAC1* gene (P29S) has been identified in melanomas by a genome-wide analysis [26,27]. After B-RAF V600 and N-RAS Q61, RAC1 P29S is the most frequent mutation found in wild-type for BRAF and N-RAS melanomas and is detected in 5–9% of all melanomas. Other frequent gain-of function mutations have been described for *RAC1* in prostate cancer (Q61), in seminomas and germ cell tumors (G12), head and neck squamous cell carcinoma (A159), cutaneous squamous cell carcinoma (P29), and lung squamous cell carcinoma (C18, P29, A159). For *RHOA*, both gain- and loss-of-function mutations have been detected in human cancers [1]. Gain-of-function mutations of *RHOA* have been described at lower frequencies in human tumors, such as RHOA A161V/P in bladder urothelial cancer and RHOA C16 and A161V/P in adult T-cell leukemia/lymphoma (see specific paragraph in this review) [28,29]. *RHOA* mutations are mainly loss-of-function mutations, such as RHOA Q5R in Burkitt Lymphoma [30,31,32] or Y42 in diffuse-type gastric carcinoma [33,34]. Indeed, mutated RHOA proteins could act as tumor suppressors in human cancers, as well as another member of the RHOA subfamily, *RHOB*, that is frequently deleted in human lung cancer [35]. Interestingly, the most frequent RHOA mutant (G17V) found in human T-cell lymphoma is a loss-of-function mutation that acts as a dominant negative mutant [36,37,38]. 

Therefore, the role of RHO GTPases in human tumors is more complex than expected and needs further investigation [1,3]. For example, it is not clear whether deregulation of RHOA GTPase pathway is sufficient for tumorigenesis or additional genetic lesions are needed for the acquisition of a full transformed phenotype [36,37,39]. Indeed, in T-cell lymphoma mutated *RHOA* is always associated with the mutations in epigenetic regulators, such as *TET2*, *DNMT3A,* and *IDH2* [37,40], and studies using animal models expressing the RHOA G17V, specifically in T cells, suggest that TET2 deletion is required to develop lymphoma [36,39] (see specific paragraph in this review). In this review we present the latest advances in the field with particular focus on the role of the most studied typical RHO GTPases such as CDC42, RAC1, and RHOA and the atypical RHOH in the initiation or progression of human lymphomas. 

## 2. Regulation of RHO Family GTPases 

The RHO family GTPases are guanine-nucleotide-binding enzymes that bind the guanosine triphosphate (GTP) and catalyze its hydrolysis to guanosine diphosphate (GDP). Over 30 years of studies have clarified their regulation and function. Their activity is tightly regulated by guanine nucleotide exchange factors (GEF), GTPase-activating proteins (GAP), and guanine dissociation inhibitors (GDI) [41]. RHO GTPases are active in the GTP-bound state and inactive in the GDP-bound state, and the ratio between GTP-bound/GDP-bound (active/inactive) conformations is critical for the proper intracellular signaling. In response to extracellular stimuli, such as mitogens or other soluble molecules that bind to the cell-surface receptors, RHO GTPases switch from an inactive GDP-bound state to an active GTP-bound state. The activation causes a conformational change of RHO GTPases and increases their ability to bind to the effector proteins and to initiate a downstream signaling cascade that in turn regulates several cellular processes depending on the stimulus and cell type. GEF proteins catalyze the exchange of GDP for GTP, thereby turning on the GTPase signaling, whereas GAP proteins increase the intrinsic GTP hydrolysis rate of the GTPase, thereby turning off the signaling. A second layer of regulation is represented by the GDI proteins that bind the GDP-bound RHO GTPase, and sequester it in the cytosol thus preventing its membrane localization or activation by GEFs [41,42,43]. RHO GTPase regulators play a crucial role in RHO GTPase activity and, interestingly, altered expression levels of GEFs, GAPs, and GDIs have been frequently described in human cancer, as well as mutations in a subset of tumors. Aberrant expression levels or mutations of GEFs, GAPs, or GDIs lead to increased activation of RHO GTPase signaling cascades that in turn promote cancer initiation and progression [15]. Indeed, several GEFs, such as ECT2, P-REX1 and P-REX2, TIAM 1, LARG and VAV family, are frequently overexpressed in human cancer [44]. For example, in luminal breast cancer the RAC-specific GEF P-REX1 is activated by upstream tyrosine kinases and G-protein coupled receptors and promotes metastasis [45], whereas mutations affecting P-REX2 have been described in melanoma [46]. The VAV family is involved in different human tumors: the hematopoietic specific VAV1 is ectopically expressed in pancreatic cancer and is correlated with poor prognosis [47] and the other members, VAV2 and VAV3, have been related to breast cancer progression [48]. Interestingly, to date, only the RHO GEF VAV1 has a role in lymphomagenesis, either as a genetic driver in T-cell lymphoma (see specific paragraph in this review) or as a downstream molecule triggered by the oncogenic kinase anaplastic lymphoma kinase (ALK) in ALK-driven anaplastic large cell lymphoma (ALCL) [24,25,49,50].

RHO GTPase downstream effectors are also important in mediating the RHO GTPase functions. Once activated, RHO GTPases bind to a variety of downstream effectors, including scaffold/adaptor proteins, kinases and actin-binding proteins. Through their downstream effectors, each RHO GTPase exerts its activity and function: RHOA stimulates stress fiber formation mainly via Rho-kinase 1 (ROCK1), whereas RAC1 and CDC42 regulate cytoskeleton and promote actin-remodeling through either p21-activated kinases (PAK1) or the Wiskott–Aldrich syndrome protein (WASP), and WASP-related WAVE, respectively. Interestingly, RAC1 and CDC42 have actin-independent activities because they can also activate the MAP kinase pathway to affect tumor cell proliferation. 

Atypical RHO GTPases show an altered GTP/GDP cycling. They are active in a GTP-bound state, but they are not strictly regulated by GEFs and GAPs [2,51]. There are two classes of atypical RHO GTPases: those that possess an elevated intrinsic guanine nucleotide exchange activity, so-called fast cycling RHO GTPases (i.e., RHOU and RHOD); and those that have an altered GTPase activity as a consequence of amino acid substitutions in the RHO domain, so called GTPase defective RHO GTPases (i.e., RHOH, RND, and RHOBTB). Thus, atypical GTPases are controlled by different mechanisms, including transcriptional regulation at a gene level or post-translational modifications, such as phosphorylation, ubiquitylation, and sumoylation.

For both typical and atypical RHO GTPases alterations in their regulation may result in a defective activity and deregulation of their downstream pathways, thereby leading to altered functions inside the cells.

### Post-Transcriptional and Post-Translational Regulation of RHO GTPases

Typical RHO GTPases can also be regulated by other mechanisms than the classical GTPase cycle. An important level of regulation is represented by post-translational lipid modifications that mediate the membrane localization of RHO GTPases and the subsequent interaction with their downstream effectors. Typically, RHO GTPases are prenylated on the cysteine (C) at their C-terminal CAAX motif (i.e., addition of farnesyl or geranylgeranyl chain) followed by a proteolytic cleavage of the terminal three residues (AAX) and methylation of the cysteine (C). This post-translational lipid modification is fundamental for translocation of RHO GTPases to the plasma membrane and is required for their biological activity [41].

Other post-translational modifications include phosphorylation and ubiquitylation. Phosphorylation can affect the normal GTP/GDP cycling thereby influencing the interaction with RHO GTPase regulators or their downstream effectors. Different kinases such as PKA, SRC, and AKT can phosphorylate RHOA, CDC42, and RAC1 [41,52]. Phosphorylation can either activate or inhibit RHO GTPase activity, although an inhibitory effect has been observed in most cases [2,41]. RHOA can be phosphorylated on Ser188 by PKA as an inhibitory mechanism because it enhances its interaction with RHO GDI and its translocation from the plasma membrane to the cytosol and decreases its interaction with the downstream effector protein ROCK1 [53,54,55]. RAC1 can be negatively regulated by phosphorylation on different residues by different kinases, such as Ser71 by AKT, Thr108 by ERK, and Tyr64 by SRC (or FAK) [56,57]. Interestingly, the Tyr64 is in the switch region 2 of RAC1 and is highly conserved in CDC42, where its phosphorylation seems to promote the binding to RHO GDIs and decrease the association with GEFs and downstream effectors [58]. The phosphorylation of the analogous residue of RHOA (Tyr66) can affect its binding to downstream effectors [59].

Ubiquitylation is an important layer of RHO GTPase regulation and is characterized by the covalent attachment of ubiquitin molecules to the lysine residues to induce proteasome-mediated degradation [41]. Thus, ubiquitylation can regulate the stability and protein levels of RHO GTPases. Ubiquitylation can affect both the active GTP-bound and inactive GDP-bound form of RHO GTPases. For example, RHOA can be ubiquitylated in different conformations and by different ubiquitin ligases (SMURF1, BACURD-CUL3, and FBXL19) depending on its conformation and in response to specific signaling [41]. RAC1 can undergo degradation only in an active GTP-bound state and this modification is somehow correlated to cancer progression. The loss of expression of RAC1 ubiquitin ligase, HACE1 E3 ligase, induces RAC1 hyperactivation and contributes to the RAC-mediated tumor progression in breast cancer [60].

RHO GTPases can undergo transcriptional regulation that can lead to alternative splicing. Indeed, RAC1b, a splice variant of RAC1 with an accelerated GDP/GTP-exchange and an impaired GTP-hydrolysis, is specifically expressed at various stages of neoplastic progression in colorectal cancer [61,62].

Moreover, several RHO GTPases can be regulated by microRNAs (miRNAs) that can either inhibit translation or degrade mRNA. Interestingly, this level of regulation has been described in cancer cells [2,63]. Same miRNA can regulate different RHO GTPases, for example miRNA-185 can affect the protein levels of both RHOA and CDC42 in colon cancer cells [64], or each RHO GTPase is a specific target; miRNA-155 specifically targets RHOA [65] and miRNA-29 targets CDC42 [66].

Despites several evidences that post-transcriptional or post-translational modifications of RHO GTPases can be altered in some human cancers, no such examples have been described so far in human lymphomas.

Overall the scenario of RHO GTPase regulation is quite complex. Deregulated post-transcriptional and post-translational modifications can lead to inappropriate RHO GTPase localization or function and contribute to tumorigenesis, as well [2,15,52].

## 3. Functional and Genetic Alterations of RHO Family of GTPases in Lymphoma

In general terms, RHO GTPases are mostly involved in human tumorigenesis as downstream effectors of driver oncogenes. However, each member of this family can have a distinct role depending on the cell and tumor type. In solid tumors, RHO GTPases are frequently activated by upstream tyrosine kinase receptors, such as ERBB2 or MET, and contribute to tumor progression and metastasis. In hematological malignancies, deregulation of the RHO GTPase signaling network has been reported, mainly related to constitutive activation of upstream signaling and implicated to tumor dissemination and invasion [14]. More specifically, in human lymphomas recurrent somatic mutations have been reported only for the GTPase RHOA and for the atypical GTPase RHOH (Table 1), whereas somatic mutations affecting RAC proteins or CDC42 have been described in solid tumors. In the following paragraphs, we describe the most recent findings on the role of RHO GTPases and their regulators in lymphoma pathogenesis.

## 4. RAC1 and CDC42 in the Pathogenesis of Lymphoma

In adult T-cell leukemia/lymphoma (ATL), RAC1 is activated by the RHO GEF TIAM1 (T-cell lymphoma invasion and metastasis) and regulates the formation of lamellipodia to enhance tumor cell infiltration [69]. In mantle cell lymphoma (MCL) RAC1 overexpression leads to its increased activity and is correlated with a shorter survival in patients [70,73]. Both RAC1 and CDC42 play a prominent role in the ALK-driven ALCL, a subtype of T-cell lymphoma [50,72]. Of note, in ALK+ ALCL they have pro-oncogenic functions because ALK promotes lymphoma cell proliferation and survival by increasing their GTPase activity through the direct phosphorylation of their RHO GEFs VAV1/VAV3. In addition, both CDC42 and RAC1 contribute to lymphoma cell dissemination in mouse models of ALCL [71]. In ALK+ ALCL the ALK-dependent downregulation of WASP contributes to the lymphoma proliferation/survival by increasing active CDC42 and MAPK pathway. This CDC42-WASP axis provided a therapeutic vulnerability because WASP deficient cells were more sensitive to MAPK inhibitors when used in combination with the ALK inhibitor crizotinib [74].

## 5. RHOA in the Pathogenesis of Lymphoma

Recent studies using whole exome sequencing technology revealed recurrent mutations of RHOA (Table 1) in different human lymphomas of both B-cell and T-cell origin, including angio-immunoblastic T-cell lymphoma (AITL), peripheral T-cell lymphoma not otherwise specified (PTCL-NOS) [37,38,40], adult T-cell leukemia/lymphoma (ATL) [25,29], diffuse large B-cell lymphoma (DLBCL), and Burkitt lymphoma (BL) [32,67]. Further studies on the biological significance of these mutations suggested a driver role for RHOA in the pathogenesis of these lymphomas.

### 5.1. Angio-Immunoblastic T-Cell Lymphoma and Peripheral T-Cell Lymphoma Not Otherwise Specified

AITL and PTCL-NOS are peripheral T-cell lymphomas (PTCL) that represent a group of aggressive non-Hodgkin lymphomas derived from mature T cells [75]. AITL is a common subtype of PTCL with unique pathological, molecular, and clinical features corresponding with the malignant transformation of T follicular helper (T_FH_) cells, whereas PTCL-NOS shows extreme cytological and phenotypic heterogeneity and lacks a specific characterization. Different studies found that 53–71% of AITL cases and 8–18% of PTCL-NOS carry the same mutation of the *RHOA* gene [25,29,37,38,40,76]. Almost all of these mutations are heterozygous and affect the glycine in a hotspot position 17 (Gly17) in the GTP/GDP-binding domain of RHOA. Structural analyses and in vitro studies highlighted the dominant-negative function of RHOA G17V mutant because this mutation impairs the binding of GTP, interferes with its own activation, and fails to activate its effector proteins. In addition, mutant RHOA affects the activity of wild-type *RHOA* because it prevents the GTP binding to wild-type RHOA by sequestering or altering the activity of the RHO GEFs. As a consequence, ectopic expression of RHOA G17V in fibroblasts reduced the formation of actin stress fibers and in a T-cell model did not induced the serum response factor pathway, known to be activated by RHOA signaling [38]. Remarkably, the presence of *RHOA* mutations in most PTCL-NOS cases correlated with a T_FH_ cell phenotype (T_FH_ cells) similar to the typical T-cell phenotype of AITL and suggested a strong association between RHOA G17V mutation and T_FH_ cell phenotype [77]. This observation is supported by in vitro and in vivo studies that report an induction of a T_FH_ cell phenotype (CXCR5^+^ PD1^+^) upon expression of G17V in CD4^+^ T cells [36,39,78]. Moreover, in two murine models, i.e., a transgenic model that express RHOA G17V under CD4 control elements [39] and a conditional knock-in model expressing the mutation G17V in the endogenous *Rhoa* locus [36], CD4^+^ T cells expressing RHOA G17V were hyper-reactive to T-cell receptor stimulation.

Interestingly, in AITL *RHOA* mutations are frequently associated with loss-of-function mutations of the *TET2* (Ten-Eleven Translocation 2) gene that encodes a protein involved in epigenetic programming and stem cell maintenance [37,38,79]. Mouse models suggest a cooperative role between TET2 and RHOA in AITL lymphomagenesis and indicate in the RHOA-dependent activation of ICOS-PI3K pathway, a targetable vulnerability for targeted therapies [36,39]. Of note, extracellular signals transduced through ICOS co-receptor is fundamental for the differentiation of naive CD4^+^ T cells into T_FH_ cells and for their proper function [80,81]. T_FH_ cells are a subtype of CD4^+^ lymphocytes generated in the germinal center that play a key role for the differentiation and survival of B cells through co-stimulatory receptors and secretion of cytokines [82]. In *Tet2^-/-^ Rhoa G17V* AITL mouse models, the expression of RHOA G17V in CD4^+^ T cells induced T_FH_ cell polarization and increased proliferation through upregulation of ICOS and activation of PI3K-AKT-mTOR and MAPK pathways [36]. Indeed, CD4^+^ T cells in mice expressing RHOA G17V showed a higher proliferation rate and a sustained activation of AKT and ERK1/2 associated with increased S6K phosphorylation [36]. Accordingly, a gene set enrichment analysis of CD4^+^ T cells from RHOA transgenic mice identified a PI3K-AKT-mTOR signaling signature suggesting that RHOA G17V mainly exerts is oncogenic activity promoting mTOR signaling [39]. These findings highlight that changes in activity of RHOA modulate the differentiation of normal T cells and act as a tumor suppressor in T cell lineage; as a consequence, its inactivation through mutations can lead to hyperactivation of oncogenic pathways and cellular transformation.

### 5.2. Adult T-Cell Leukemia/Lymphoma

ATL is an aggressive form of PTCL caused by the human T-lymphotropic virus type 1 (HTLV-1) [75]. Recurrent mutations in the *RHOA* gene have been reported in 15% of ATL patients [25,29]. Unlike the pattern of mutations described in AITL and T_FH_-like PTCL-NOS that are mainly located in the hot spot G17V, mutations in ATL span the entire coding region of *RHOA* [29]. However, the majority is invariably located in the GTP-binding domain where the position Cys 16 (C16R) was the most frequently observed. The RHOA mutant A161E did not bind GTP and is biochemically inactive similarly to the G17V mutant detected in AITL and T_FH_-like PTCL-NOS. Surprisingly, other RHOA mutants (C16R and A161P) revealed different or even opposite functional consequences as they showed fast GTP/GDP cycling and increased RHOA activity [29]. Therefore, in ATL either loss- or gain-of-function RHOA mutants are involved in ATL lymphomagenesis. ATL can arise from different subsets of CD4^+^ T cells: naive, activated and regulatory T cells, and RHOA mutations seem to be associated with the different phenotypes. ATL cells with activating C16R and A161P mutations have Treg or effector T-cell phenotype, whereas ATL cells with inactivating G17V have memory T-cell phenotype. Therefore, the functional difference of the cell of origin can explain the occurrence of RHOA mutations in ATL. Alternatively, the critical role of RHOA in TCR signaling and T-cell development and differentiation can explain this discrepancy. Indeed, deregulation of the RHOA activity can trigger different downstream signaling network inducing the cellular differentiation of T cells into Treg/effector T-cell or memory T-cell lineages.

### 5.3. Diffuse Large B-Cell Lymphoma and Burkitt Lymphoma

*RHOA* mutations have also been found in lymphomas of B-cell origin. Genomic studies on Burkitt lymphoma and DLBCL have reported a frequency of *RHOA* mutations of 7–9% and <5%, respectively [32,67]. These mutations are mostly located in position Arg5 (R5Q) and to a lesser extent in positions Tyr42 in the switch 1 region and Leu69 in the second GTP-binding domain. Mutations that affect the GTP-binding domain generate inactive proteins unable to bind GTP and interact with RHO GTPase regulators (GEFs and GAPs). They also inhibit the activity of the RHOA wild-type as described for the RHOA G17V mutant in AITL. Mutations in the switch/effector region (Y42F/H/S) affect the interaction with downstream effectors and deregulate the downstream signaling. These are loss-of-function mutations because they suppress RHOA activity, but it is not clear how they promote tumorigenesis in BL and DLBCL. Remarkably, in BL and DLBCL RHOA is also inactivated by mutations that affect the upstream molecules of the Gα13-dependent pathway [83]. This pathway controls the confinement of B cells within the B-cell follicle and its deregulation alters germinal center B-cell migration and is associated with lymphomagenesis. Mutations located in genes of the Gα13-dependent pathway, including sphingosine-1-phosphate receptor 2 (S1PR2) and P2Y purinoceptor 8 (P2RY8), GNA13 (which encodes Gα13), and RHO guanine nucleotide exchange factor 1 (ARHGEF1) are found in both GCB-DLBCL (30%) and Burkitt lymphoma (15%) [84]. Studies in mouse models deficient for S1PR2 or Gα13 have shown that they develop tumors with features of GCB-DLBCL and with constitutive activation of the AKT pathway [83,85]. In these mice loss of the Gα13 pathway resulted in disruption of the GC architecture and dissemination of GC B cells to distant sites as observed in DLBCL. Disruption of this pathway impacts on downstream RHOA functions by suppressing its activity. Overall RHOA and the axis Gα13/RHOA have suppressive functions in DLBCL and BL because their suppression or inactivation by mutations promotes lymphomagenesis.

Overall, even if historically RHOA was described as a molecule with pro-oncogenic functions because it is frequently overexpressed in human tumors and associated with tumor progression, recent findings on RHOA mutations describe a more complex and heterogeneous landscape, at least in human lymphoma. Both gain-of-function and loss-of-function mutations, as well as dominant-negative mutations, with contrasting functions have been reported in lymphoma and the strong correlation between the tumor type and the mutational distribution indicates that the functional consequence of a *RHOA* mutation during lymphoma development depends on the cell of origin, as observed in ATL. Further biological studies with appropriate in vitro and in vivo models will help to elucidate the functional significance of gain- and loss-of-function mutations of *RHOA* in human lymphoma.

## 6. RHOH in the Pathogenesis of Lymphoma

RHOH belongs to the “atypical” RHO GTPase family [6,86]. RHOH is regulated by post-translational modifications while remaining constitutively active because it lacks intrinsic GTPase activity. Physiologically, RHOH is expressed in hematopoietic cells and, in particular, is highly expressed in T lymphocytes. RHOH is activated through tyrosine phosphorylation of its ITAM-like motif upon TCR engagement and mediates the recruitment of ZAP70 and LCK to the immunological synapse. In RHOH knock-out mice, RHOH deficiency affects T-cell development in the thymus, impairs T-cell function, and causes peripheral T-cell lymphopenia [87,88,89].

*RHOH* was first identified in a chromosomal translocation, t(3;4)(q27;p23), with the *LAZ3/BCL6* gene in a non-Hodgkin lymphoma cell line and in multiple melanoma patient within a translocation involving the *IgH* gene, t(4;14) (p13;p32) [90]. Despite these observations, the role of RHOH in the pathogenesis of these tumors has not yet been established. However, in lymphomas alterations of RHOH expression and activity are more frequently linked to the presence of somatic hypermutations in the non-coding region than chromosomal rearrangements. Indeed, somatic hypermutations of RHOH occur at high frequency (46%) in diffuse large B-cell lymphoma (DLBCL), an aggressive subtype of non-Hodgkin lymphoma, but do not have prognostic significance [68,86]. Interestingly some studies reported accumulation of aberrant somatic hypermutations in *RHOH* and other genes during disease progression, such as the transformation of follicular lymphoma and chronic lymphocytic leukemia to DLBCL [86]. Nonetheless, a direct correlation with overall survival or disease-free survival was not found in a larger cohort of patients [91]. Downregulated expression of RHOH has been reported in some cases of leukemia (hairy cell leukemia and acute myeloid leukemia) although the molecular mechanisms are not yet unveiled [92,93]. Thus, despite its important role in T-cell development, recurrent genetic alterations of the *RHOH* gene have not yet been found in T-cell malignancies. Conversely, genetic alterations of RHOH have been detected in B cell lymphomas, but a direct role in their pathogenesis has not been clearly defined. In a murine model of DLBCL (Iµ-HA-BCL6 transgenic mice) [94], the deletion of *Rhoh* (crossing with *Rhoh^-/-^* mice) accelerated lymphoma progression and correlated with early death. In addition, data generated in lymphoma cells derived by murine Bcl6-*Rhoh* ko Tg mice suggested that RHOH can be involved in DLBCL development by regulating BCL6 expression [95]. Overall, a clear signaling network related to RHOH deregulation in human lymphomas is still missing and needs further studies.

## 7. Alterations of RHO GTPases Regulators and Downstream Effectors in Lymphoma

Given the activating functions of the RHO GEFs and RHO effectors in the RHO GTPase network signaling, they are typically associated with pro-tumorigenic functions in tumors, whereas RHO GAPs, that are negative regulators, are frequently considered as tumor suppressors. However, the findings in the last decade on human tumors suggest a more complex scenario where the role of GEFs and GAPs is not so clearly defined [3].

Several RHO GEFs (~70 members) and RHO GAPs (~80 members) have been identified, exceeding the 20 RHO GTPases described so far, suggesting that they can regulate each RHO GTPase depending on the cellular context [6]. RHO GEFs are mainly divided into two families based on the presence of a functional domain: DBL-homology (DH) domain family and Dock homology region (DHR) domain family [44]. Most GEFs belong to the DH family and are characterized by the catalytic DBL-domain, that catalyzes the exchange of GDP for GTP, and the pleckstrin-homology (PH) domain, that mediates the plasma membrane localization of the RHO GTPase. Among GEFs, the RHO GEF VAV1, a member of the DH family, is specifically expressed in hematopoietic cells. In particular, VAV1 is an essential mediator of the T-cell receptor (TCR) signaling in T lymphocytes and contributes to the cytoskeleton rearrangement, T-cell activation, proliferation, and survival upon TCR engagement. Recurrent activating mutations (Table 1) and chromosomal translocations in the gene encoding for VAV1 have been described in PTCL, specifically in ATL, PTCL-NOS, and ALCL [24,25,49]. Chromosomal rearrangements involving *VAV1* break the gene in the same region (loss of the C-terminal SH3 domain) that is responsible for the auto-inhibition of VAV1. The result is a novel fusion oncoprotein that lacks this domain and thereby shows constitutive activation. Somatic mutations of *VAV1* have been found in some cases of ATL and PTCL, and consistently are clustered in the same C-terminal SH3 domain, in the PH domain and in the zinc finger domain [24,49]. Overall, both chromosomal rearrangements and mutations of *VAV1* lead to the constitutive activation of VAV1 effector pathways, including the RHO GTPase RAC1, supporting a driver role for VAV1 in the pathogenesis of PTCL.

RHO GAPs and GDIs are negative regulators of the RHO GTPases, but to date no recurrent mutations or genetic aberrations have been described in human lymphoma. The GAPs DLC1 (deleted in liver cancer-1) and P190RHOGAP are frequently downregulated in human tumors as a consequence of genetic deletions or epigenetic silencing and contribute to tumorigenesis via deregulation of RHOA or CDC42 signaling network [96,97]. ARHGAP8 is found overexpressed in colon cancer indicating that deregulation of these pathways can be more complex than expected [98]. Regulation of expression of RHO GDIs has been reported in different studies, although the situation is more complex because these changes depend on the tumor type and correlate differently with the tumor phenotype. In pancreatic cancer the upregulation of GDI2 correlated with invasiveness [99], whereas in bladder cancer the downregulation of GDI2 was associated with a metastatic phenotype [100]. In addition, in studies on breast cancer conflicting results on the expression of GDI1 have been reported so far [101,102].

## 8. Targeting RHO GTPases and Their Signaling Network

Efficient and specific targeting of the small GTPases has been one of the major goals in cancer research but it has not been accomplished yet for different reasons, a situation that is reminiscent of the challenges of targeting mutated K-RAS [103]. First, GTPases are small molecules with few targetable domains and limited druggability of their catalytic domains. Second, the low concentration of GTP within the cell and the high binding affinity with the RHO GTPase, even at very low concentrations, has prevented to effectively inhibit GTPases with nucleotide analogs. Third, the unavoidable occurrence of toxic side effects of some RHO GTPase inhibitors as a consequence of their broad range of action. Last, the lack of knowledge about the role of each GTPase in human cancer, i.e., the recent description of a tumor suppressor activity for RHOA in AITL, PTCL, and Burkitt’s lymphoma, has raised concerns about a strategy of inhibition of RHO GTPase signaling. 

Overall, traditional drug design approaches have not worked so far, and few advances have been made in this field [104,105]. Most of the current drugs aim at disrupting the interaction between the RHO GTPase and its GEF, thereby blocking its activity. Some of them have been tested at preclinical levels and showed specific activities: Rac1 inhibitor NSC23766 disrupts the interaction of RAC1 with its GEF TIAM1 and does not influence the binding of CDC42 or RHOA with their GEFs; the small molecule CASIN specifically inhibits CDC42 and its GEF interaction without affecting other Rho GTPase activity. A derivative of NSC23766, EHop-16, has shown higher efficacy than NSC23766. It inhibits both RAC1 and CDC42 by interfering with the interaction with the RHO GEF VAV1. Non-competitive inhibitors, such as ML141 for CDC42 and EHT 1864 for RAC, have been also developed to induce the dissociation of the bound nucleotide and to lock the GTPase in an inactive conformation [106,107].

Alternative strategies consist in pharmacologically modulating the activity of the upstream regulators RHO GEFs and RHO GAPs or the downstream effectors. However, this is a challenging approach and so far a poorly successful approach, as GEFs and GAPs are multi-domain and multifunctional molecules with different activities inside cells not strictly limited to RHO GTPase signaling. In addition, RHO GAPs are less appealing for the development of inhibitors because in cancer they are often associated with the loss-of-function mutations and act as tumor suppressors. For GEFs, the best strategy is to prevent the binding of a GEF with its specific Rho GTPase, thus suppressing the GTPase activity [108]. This is the case of the RHOA and the leukemia-associated RHO GEF (LARG). Based on a virtual screening using published protein:protein interactions, two different chemical probes, Y16 and Rhosin, have been generated for the inhibition of RHOA-LARG interaction without effects on other DBL family of RHO GEFs. In addition, they also have specificity for RHOC, thus inhibiting RHOC signaling as well, and preventing by-pass signaling [109]. However, none of these molecules has been tested in clinical trials to date. 

Another critical target for the inhibition of the RHO GTPase intracellular network is the downstream effector ROCK that has been a preferential target of several small molecules tested through the years. The ROCK inhibitor fasudil was initially developed in the 1980s as an intracellular calcium agonist and only later was discovered as an effective inhibitor of serine-threonine kinases, including ROCK [110]. This inhibitor binds the ATP-binding pocket of ROCK and inhibits its kinase activity. Fasudil is the only clinically approved drug for the inhibition of the RHO GTPase pathway and is used to treat pulmonary and cerebral hypertension in Japan. Recent studies on animal models of human tumors have revealed that fasudil blocks the invasion and metastasis and can interfere with leukocyte recruitment, thereby suggesting its use in cancer treatment. A compound with similar properties, Y-27632, was developed and used in vivo to study the regulation of cytoskeleton in inflammatory diseases but it has not yet been tested in clinical trials [111,112]. Other compounds derived from Y-27632 have been successfully tested for the inhibition of lung metastasis formation in animal models and one of them, ripasudil, is currently approved for the treatment of glaucoma in Japan [113]. 

Another downstream effector of RHO GTPases that has been extensively studied to find specific drugs is the CDC42 and RAC1 effector PAK1 [64,114]. Several ATP competitive and non-competitive inhibitors have been developed but none of them have reached the clinics for several reasons, such as poor specificity, high toxicity, and chemical instability. However, some of these compounds specifically developed as anti-cancer drugs have shown great efficacy in animal models, thereby suggesting therapeutic promise.

Thus, albeit no drugs that target RHO GTPase pathway are currently in clinical trials for cancer therapy, the members of the RHO GTPase family are still considered interesting targets for the development of innovative cancer treatments due to the central role they play in many cellular processes. Overall, the main challenge remains to find compounds able to interfere with the key RHO GTPase interaction and not just to inhibit the RHO GTPase network as a whole.

## 9. Conclusions

Different genetic and epigenetic mechanisms can deregulate the RHO GTPase signaling network in lymphomas and, in general, in human tumors; the variability of alterations found in different tumors reflects the complex regulation of this signaling in normal cells. These mechanisms include activating or inactivating mutations of the RHO GTPases or of their regulating proteins as well as changes in expression levels that altogether generate an aberrant signaling with pro- or anti-tumorigenic functions depending on the cell context and tumor type. Unraveling these networks will enable the development of compounds that could be therapeutically effective.

## Figures and Tables

**Table 1:** Recurrent genetic lesions affecting RHO GTPase network in human lymphomas. Highlighted in bold are the most frequent mutants.

RHO GTPase	Mutations	Functional Consequence	Tumor type	Frequency	References
RHOA	**G17V** A161E	Loss-of-function	T_FH_-like PTCL-NOS	8–18%	[37]
			AITL	53–71%	[37,38,40]
	**C16R**/F G17V G14V A161P/V K118E/Q	Gain-of-function Loss-of-function Gain-of-function Gain-of-function Gain-of-function	ATL	15%	[29]
	**R5Q**/W Y42F/H/S	Loss-of-function	DLBCL	<5%	[67]
	**R5Q**/W Y42F/H/S	Loss-of-function	BL	7–9%	[30,31,32]
RHOH	Somatic hypermutations	Deregulation of BCL6 expression	DLBCL	46%	[68]
VAV1	VAV1-GSS VAV1-MYO1F VAV1-S100A7 VAV1-THAP4	Constitutive activation	PTCL-NOS	11%	[24,49]
	VAV1-GSS	Constitutive activation	ALCL	11%	[24]
	**E556D/K** E175V/L Y174C K404R D797N/H R798P/Q R822Q/L	Gain-of-function	ATL	18%	[25]
	VAV1 Δ778–786	Constitutive activation	PTCL-NOS	ND	[49]
RAC1	WT	Hyperactivation	ATL	ND	[69,70]
RAC1 and CDC42	WT	Hyperactivation	ALK+ ALCL	ND	[50,71,72]
